# A Sustainable Air-Assisted Liquid–Liquid Microextraction Procedure Coupled to Liquid Chromatography–Tandem Mass Spectrometry for the Determination of 11-Nor-9-carboxy-Δ^9^-tetrahydrocannabinol and Hexahydrocannabinol Metabolites in Urine

**DOI:** 10.3390/jox16040143

**Published:** 2026-08-01

**Authors:** Pamela Cabarcos-Fernández, Sara Odoardi, Serena Mestria, Valeria Valentini, Giulia Biosa, Ana María Bermejo-Barrera, Sabina Strano-Rossi

**Affiliations:** 1 Forensic Toxicology Service, Institute of Forensic Science, Faculty of Medicine, Universidad de Santiago de Compostela, C/San Francisco s/n, 15782 Santiago de Compostela, Spain; pamela.cabarcos@usc.es; 2 Forensic Toxicology Laboratory, Department of Health Surveillance and Bioethics, Università Cattolica del Sacro Cuore Fondazione Policlinico Gemelli IRCCS, 00168 Rome, Italy; sara_odoardi@unicatt.it (S.O.); serena.mestria@unicatt.it (S.M.); valeria.valentini@unicatt.it (V.V.); giulia.biosa@unicatt.it (G.B.); sabina.stranorossi@unicatt.it (S.S.-R.)

**Keywords:** hexahydrocannabinol, metabolites of HHC, urine, air-assisted liquid–liquid microextraction, LC-MS/MS

## Abstract

Hexahydrocannabinol (HHC) is a semi-synthetic cannabinoid that has recently emerged in the European market, raising concerns regarding its detection in forensic toxicology. Reliable analytical approaches are required for the determination of HHC metabolites together with conventional cannabis biomarkers in biological samples. This study aimed to develop and validate a sample preparation method based on air-assisted liquid–liquid microextraction (AALLME) coupled to liquid chromatography–tandem mass spectrometry (LC–MS/MS) for the determination of 11-nor-9-carboxy-Δ^9^-tetrahydrocannabinol (THC-COOH) and HHC metabolites in urine. Urine samples underwent alkaline hydrolysis followed by AALLME using a cyclohexane/ethyl acetate mixture (9:1) prior to LC–MS/MS analysis. Experimental conditions affecting extraction performance were optimized. Method validation was performed according to international guidelines. The method achieved a limit of detection of 2.5 ng/mL and a lower limit of quantification of 5 ng/mL. Precision and accuracy fulfilled the established acceptance criteria across all concentration levels. The applicability of the method was evaluated using eleven authentic THC-COOH-positive routine casework urine samples. THC-COOH was successfully quantified, whereas no HHC metabolites were detected because no urine samples from confirmed HHC users were available. The proposed workflow provides a reduced-solvent sample preparation approach compared with conventional procedures and demonstrated adequate analytical performance for the simultaneous determination of THC-COOH and HHC metabolites in urine.

## 1. Introduction

In 2022, a new group of NPS (New Psychoactive Substances) with cannabinoid-like effects appeared on the recreational market, with hexahydrocannabinol (HHC) being the first to be identified in Europe. These compounds are structurally similar to Δ^9^-tetrahydrocannabinol (Δ^9^-THC), the primary psychoactive component of *Cannabis sativa* L. The earliest examples were synthesized from Δ^9^-THC or cannabidiol (CBD), which is why they became known as semisynthetic cannabinoids [1]. Over time, this term has also been extended to include other cannabimimetic substances that, although not directly derived from Δ^9^-THC or CBD, share comparable structural characteristics [2,3,4]. Initially, these substances are not listed in the United Nations Convention on Psychotropic Substances [5], which means they are not illegal. They are commercially available in various forms, including low- Δ^9^-THC cannabis flower and resin that have been sprayed or infused with HHC, vaping products, and edible formulations. Notably, HHC-treated plant material closely resembles illicit cannabis in both appearance and odour, raising concerns about its potential to be deliberately or unintentionally misrepresented as conventional cannabis or used to adulterate Δ^9^-THC and CBD-containing products [1,4,6,7].

To demonstrate cannabinoid consumption, metabolites are typically used as reliable analytical targets in urine testing, since the original cannabinoids are often either not present or found only in low concentrations. At present, little information is available on the pharmacokinetics and metabolism of HHC in humans and animals [1,4]. Various studies have identified hydroxylated metabolites in urine samples, including 11-hydroxy-9(R)-hexahydrocannabinol (11-OH-9(R)-HHC), 8-hydroxy-9(R)-hexahydrocannabinol (8-OH-9(R)-HHC) and 11-nor-9-carboxy-9(R)-hexahydrocannabinol (HHC-COOH) [2,3,8,9,10,11]. The literature reviewed emphasizes the need for further research to better understand the metabolic profile of these compounds. Similarly, studies by Patton A. [12], Muir L. [13], and Grapp M. et. al. [14] suggest that additional research is required to determine whether HHC-COOH may be a metabolite of Δ^9^-THC rather than solely a product of HHC consumption.

Several studies have highlighted the need to establish new confirmatory methods in toxicological analysis laboratories for the identification and quantification of semisynthetic cannabinoids [3,6,15,16]. This need arises from the occurrence of false-positive results associated with immunoassay techniques, as observed in several European countries where the presence of these compounds has been detected [2,3,17]. The WHO (World Health Organization) also highlights the issue of false positives caused by the presence of HHC in its Critical Review Report Hexahydrocannabinol [18]. These inaccuracies are largely attributed to the structural similarity between semisynthetic cannabinoids, Δ^9^-THC, and its main metabolite, THC-COOH. Therefore, cannabis drug testing cannot rely solely on immunoassay screening methods, as these lack the specificity required to distinguish between different tetrahydrocannabinol and hexahydrocannabinol analogues, some of which are controlled substances while others remain unregulated. Several studies have reported the use of more robust analytical techniques for the detection of HHC and its metabolites, including gas chromatography–mass spectrometry (GC–MS), high-performance liquid chromatography coupled to high-resolution tandem mass spectrometry (HPLC–HRMS/MS), and liquid chromatography–tandem mass spectrometry (HPLC–MS/MS) [19]. Nevertheless, these approaches still present important limitations, such as inadequate sensitivity in certain cases, prolonged analysis times, the requirement for derivatization steps, and the need for highly specialized personnel.

**Figure 1:**
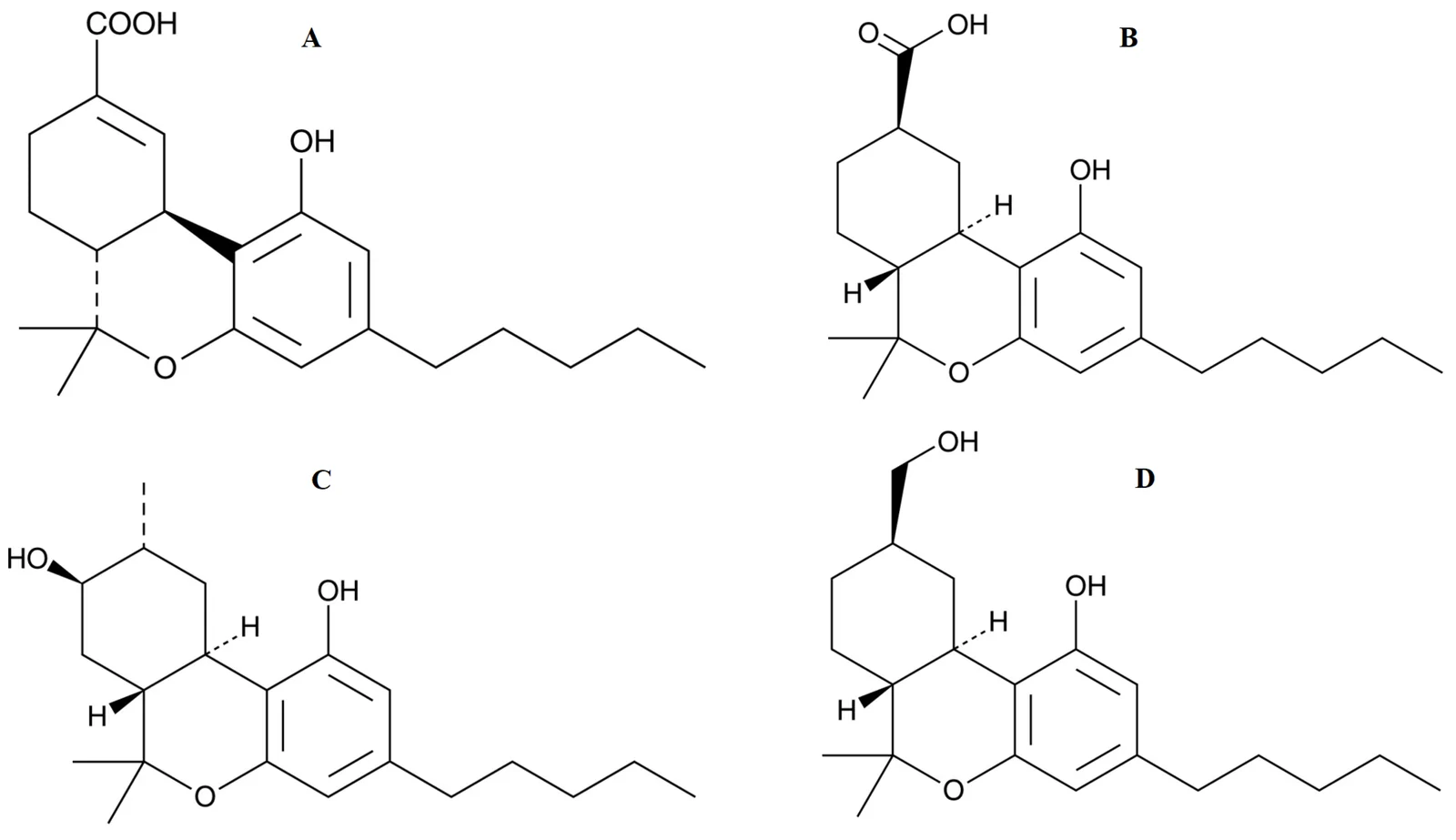
Chemical structures of the compounds studied ((**A**) 11-nor-9-carboxy-Δ^9^-tetrahydrocannabinol (THC-COOH); (**B**) 11-nor-9-carboxy-9(R)-hexahydrocannabinol (HHC-COOH); (**C**) 8-hydroxy-9(R)-hexahydrocannabinol (8-OH-9(R)-HHC); (**D**) 11-hydroxy-9(R)-hexahydrocannabinol (11-OH-9(R)-HHC)).

Regarding extraction techniques, Muir L. et al. [13] have employed liquid–liquid extraction (LLE) and solid-phase extraction (SPE) for the isolation of semisynthetic cannabinoids, whereas Di Trana D. et al. [19] applied the QuEChERS approach. To date, no studies have reported the application of air-assisted liquid–liquid microextraction (AALLME) for the determination of HHC metabolites in urine. Compared with conventional extraction procedures such as LLE and SPE, AALLME requires only a small volume of extraction solvent, eliminates the need for a disperser solvent by achieving dispersion through repeated aspiration cycles, and represents a simplified sample preparation approach with reduced organic solvent consumption. The objective of the present work was not to replace established extraction procedures, but to evaluate whether AALLME could provide a simple extraction approach requiring low organic solvent consumption for the simultaneous determination of THC-COOH and HHC metabolites (see Figure 1 for chemical structures) in urine, while maintaining satisfactory analytical performance. To the best of our knowledge, this is the first report describing the application of AALLME for the simultaneous determination of these metabolites in urine samples. Although AALLME has been widely applied in environmental analysis, its use in forensic toxicology remains limited [20,21,22,23,24,25,26,27].

Therefore, the presented study aimed to identify and quantify THC-COOH and some metabolites of HHC in urine (11-OH-9(R)-HHC, 8-OH-9(R)-HHC and HHC-COOH) using AALLME and LC-MS/MS. A validated method was then applied to actual cases testing positive for THC-COOH. This method may be used to quantify potential future cases testing positive for HHC metabolites, besides THC carboxy-metabolites.

## 2. Materials and Methods

### 2.1. Reagents and Chemicals

The analytical standards of the 11-hydroxy-9(R)-hexadrydrocannabinol (11-OH-9(R)-HHC), 8-hydroxy-9(R)-hexadrydrocannabinol (8-OH-9R-HHC), 11-nor-9-carboxy-9(R)-hexahydrocannabinol (HHC-COOH) were obtained from Comedical as part of the “NPS-LABVEQ” project (Comedical S.r.l., Trento, Italy), while 11-nor-9-carboxi-Δ^9^-tetrahydrocannabinol (THC-COOH) and the internal standard (IS) 11-nor-9-carboxi- Δ^9^-tetrahydrocannabinol-d3 (THC-COOH-d3) were supplied by Lipomed AG (Arlesheim, Switzerland) through Chebios S.r.l. (Rome, Italy). THC-COOH-d_3_ was selected as the internal standard because isotope-labelled analogues of the investigated HHC metabolites were not available in our laboratory at the time of the study.

All standards were available as 0.1 mg/mL solutions in methanolic solution. Working standard solutions were prepared in methanol.

Sodium hydroxide, formic acid, methanol (LC-MS grade, purity ≥99.9%), ammonium formate (>99.0%), MilliQ water, cyclohexane (ACS reagent, ≥99%), and ethyl acetate (reagent grade, ≥99.5%) were purchased from Merck (Darmstadt, Germany).

### 2.2. Urine Samples

Blank urine samples were voluntarily provided by healthy laboratory researchers. All samples were anonymized prior to analysis and were confirmed to be drug-free before use. The samples were used exclusively for analytical method development and validation, and no personal identifying information was collected. The samples were stored at +4 °C during the study to maintain sample integrity throughout the experimental workflow. Routine casework urine samples positive for THC-COOH were used to demonstrate the application of the method.

### 2.3. AALLME Sample Preparation

Aliquots of 0.1 mL urine were used for analysis, spiked with THC-COOH-d3 (15 µL Sol. 0.1 µg/mL). A basic hydrolysis was carried out using sodium hydroxide (NaOH) (25 µL of 10 M solution) on a thermostatic plate at 40 °C for 10 min. Next, 25 µL of formic acid was added, and the AALLME was carried out using the following procedure.

200 µL of a cyclohexane/ethyl acetate (9:1) mixture was added to the hydrolyzed sample. Dispersion was achieved by aspirating the mixture eight times using a Pasteur pipette. The mixture was left to settle for approximately 10 min before collecting the organic phase from the upper layer. It was then evaporated under a stream of nitrogen. The dry extract was reconstituted with 100 µL of methanol, and a 10 µL aliquot was injected into the LC-MS/MS system.

Alkaline hydrolysis was included as a preliminary sample preparation step to deconjugate urinary cannabinoid metabolites prior to extraction. The effectiveness of this treatment was qualitatively assessed in authentic urine samples by monitoring the absence of residual THC-COOH-glucuronides after hydrolysis using the routine laboratory analytical workflow. No residual signals corresponding to this glucuronide were detected under the selected hydrolysis conditions.

### 2.4. Instrumentation

**Figure 2:**
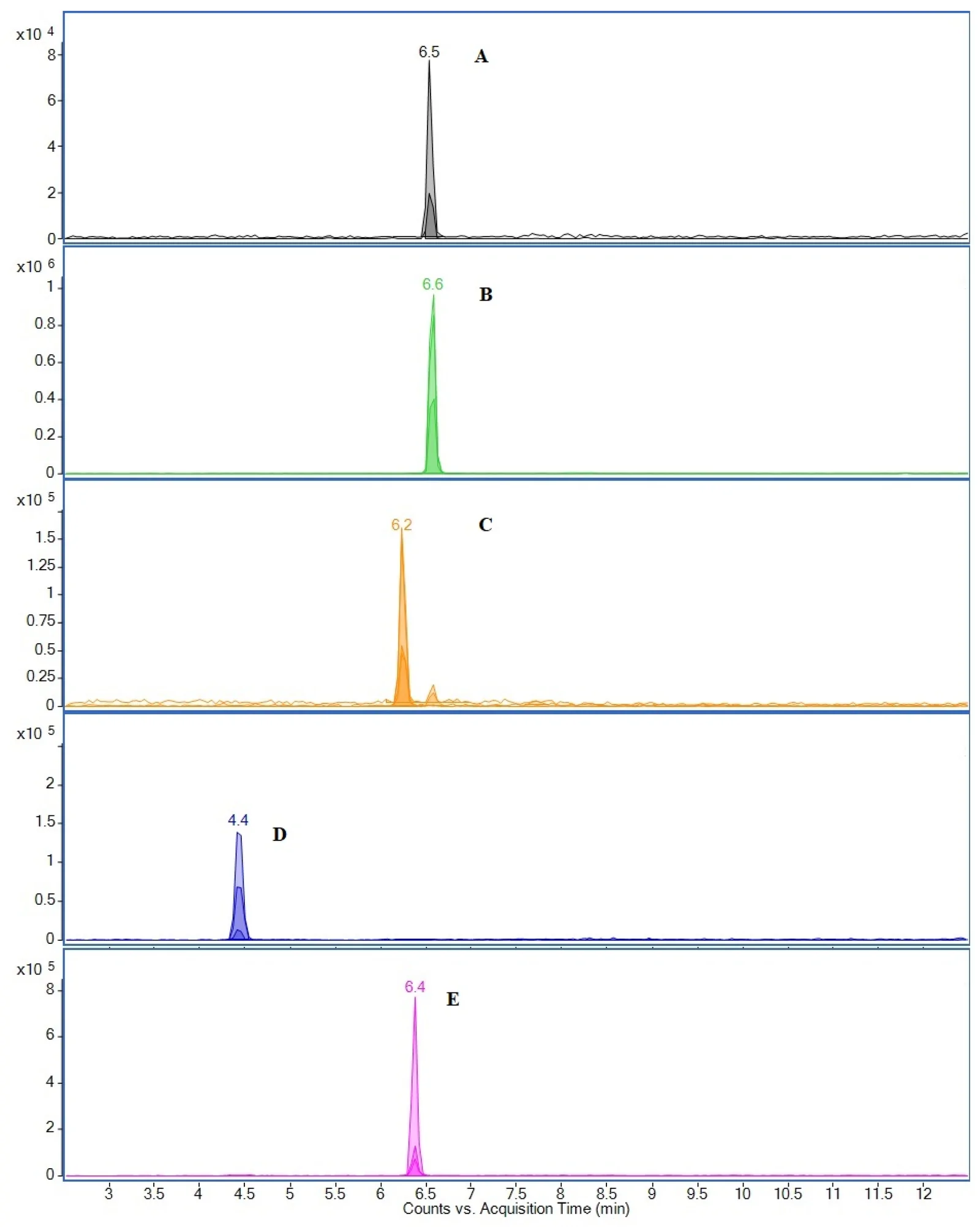
Chromatogram of semisynthetic cannabinoids ((**A**) THC-COOH-d3; (**B**) THC-COOH; (**C**) HHC-COOH; (**D**) 8-OH-9(R)-HHC; (**E**) 11-OH-9(R)-HHC).

LC-MS/MS measurements were performed with an Agilent 6460 triple quadrupole mass spectrometer (Agilent Technologies, Santa Clara, CA, USA) equipped with a Jet Stream electrospray ionization source operated in positive ion mode (ESI +), with a binary pump with integrated vacuum degasser (Agilent 1290 Infinity system), a high-performance well-plate autosampler, and a thermostatic column module. A Raptor C18 2.7-μm particle diameter reverse phase column (100 mm length × 2.10 mm i.d.) from Restek (Milan, Italy) was used for chromatographic separations. LC system was operated in gradient mode with mobile phase A being 0.1% formic acid and ammonium formate 5 mM in ultrapure water and mobile phase B 0.1% formic acid in methanol. The flow rate was set at 0.35 mL/min. Table 1 shows the gradient used. After completion of the gradient programme, a post-run equilibration time of 3 min under initial mobile phase conditions was applied before the next injection. These conditions provided optimal separation of the cannabinoids studied (Figure 2). Data acquisition was performed in multiple reaction monitoring (MRM) mode. The transitions for all compounds are shown in Table 2.

### 2.5. Method Validation

The chromatographic acquisition method used in this study was based on a previously validated LC–MS/MS procedure routinely employed in our laboratory for cannabinoid analysis. The method was expanded by incorporating the MRM transitions corresponding to the HHC metabolites investigated in the present work, while maintaining the original transitions used for routine cannabinoid testing, including those of THC-COOH-glucuronide. These glucuronide transitions were qualitatively monitored in authentic urine samples following alkaline hydrolysis as an internal control of the deconjugation step. No residual signals corresponding to THC-COOH glucuronides were detected under the selected hydrolysis conditions. Since glucuronide reference standards for the investigated HHC metabolites were not available, their corresponding MRM transitions were not incorporated into the analytical method, and no direct assessment of their deconjugation could be performed.

The validation study was performed according to Food and Drug Administration (FDA) guidelines [28] and American National Standards Institute/Academy Standards Board (ANSI/ASB) Standard 036 [29] in terms of selectivity, linearity, limit of detection (LOD), lower limit of quantification (LLOQ), intra-day and inter-day precision and accuracy tests, recovery, process efficiency and matrix effect.

Sensitivity was assessed by determining the LOD and LLOQ. The LOD was defined as the lowest concentration at which the analyte could be reliably detected according to the routine identification criteria of the analytical method. The LLOQ was established as the lowest concentration that fulfilled the predefined acceptance criteria for quantitative analysis, including acceptable precision (CV ≤ 20%) and accuracy within ±20% of the nominal concentration, in accordance with the FDA guidelines.

Selectivity of the method was demonstrated by analyzing six blank urine samples from multiple sources to confirm that the assay is free of potential interfering substances.

Calibration curves were created using a blank urine sample spiked with the studied analytes. The *y*-axis represented the ratio between the peak areas of the analytes and the IS, while the *x*-axis represented the semisynthetic cannabinoid concentration.

To assess intra- and inter-day accuracy and precision, the BIAS (%) and coefficient of variation (CV, %) have been calculated, respectively. BIAS is defined as the difference between the average results obtained using a specific analytical method and the established reference value. Precision is defined as the degree of agreement between results obtained from repeated analyses of a homogeneous sample under predefined conditions. The evaluation was performed at four concentration levels: the LLOQ, as well as low, medium, and high concentrations, with five replicates analyzed at each level. For data interpretation, acceptance criteria of ±20% were applied at the LLOQ and ±15% for the remaining concentration levels.

Extraction recovery, process efficiency, and matrix effect were evaluated to assess the performance of the proposed sample preparation procedure. Extraction recovery and process efficiency were determined to evaluate the efficiency and reproducibility of the extraction process, whereas matrix effect was assessed to identify potential ionization enhancement or suppression caused by urine matrix components. Three sets of samples (analytes prepared in neat solvent; urine samples spiked with analytes before extraction; and urine samples spiked after extraction) at three concentration levels were analyzed in quintuplicate, following the procedure described by Matuszewski et al. [30].

## 3. Results and Discussion

The following sections present the optimization and full validation results of the proposed method, which has also been designed in accordance with green chemistry principles. The microextraction approach employed is characterized by low solvent consumption and reduced environmental impact. The optimization of all experimental parameters associated with the selected method was performed step by step. Each experimental condition evaluated during method optimization was analyzed in four independent replicates (n = 4), and data are reported as mean ± SD (standard deviation). The variables investigated included the type and volume of the extraction solvent, the number of extraction cycles, and the ionic strength.

### 3.1. AALLME Optimization

The different optimized parameters are shown below.

#### 3.1.1. Study of the Extraction Solvent Type

**Figure 3:**
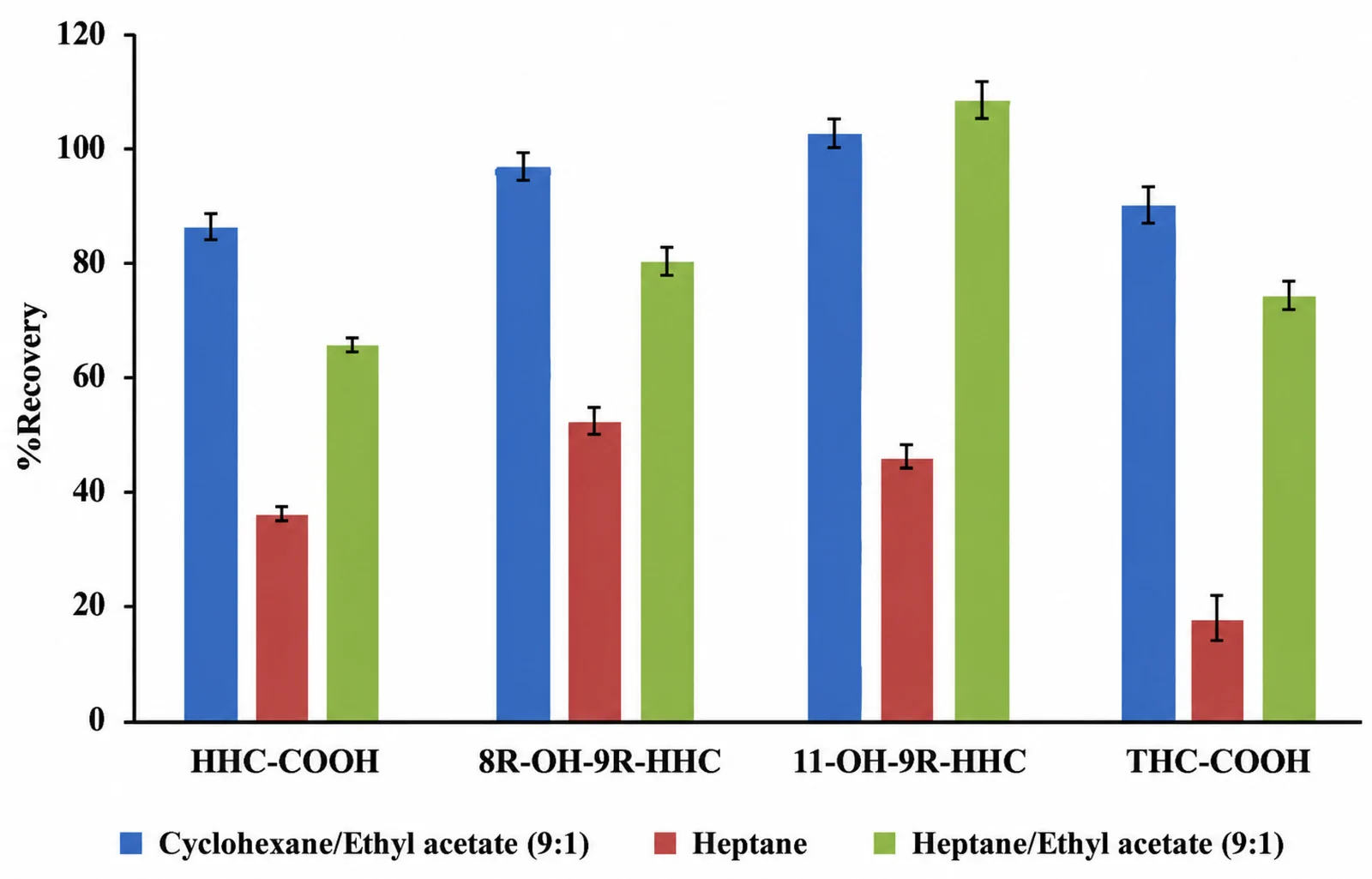
Optimization of solvent type (Fixed variables: 100 µL urine, 300 µL extraction solvent, 6 air-assisted extraction cycles, without NaCl). Data are expressed as mean ± SD (n = 4).

Various solvents were tested, with the aim of avoiding chlorinated solvents due to their higher toxicity. The solvents selected were cyclohexane/ethyl acetate (9:1), heptane and heptane/ethyl acetate (9:1). The results obtained can be seen in Figure 3. The pattern is repeated for all the compounds studied, with better recoveries obtained using cyclohexane/ethyl acetate (9:1), except for 11-OH-9(R)-HHC, where the heptane/ethyl acetate mixture appears to perform slightly better than the previous mixture. This figure demonstrates the need to add a polar component, such as ethyl acetate, to improve extraction due to the carboxylated compounds.

The choice of cyclohexane/ethyl acetate (9:1) is consistent with previous work by the researchers [26]. Furthermore, the hexane/ethyl acetate mixture in a 9:1 ratio is widely used in forensic toxicology for the simultaneous extraction of neutral and acidic compounds [31,32,33,34].

#### 3.1.2. Study of the Volume of Extraction Solvent

**Figure 4:**
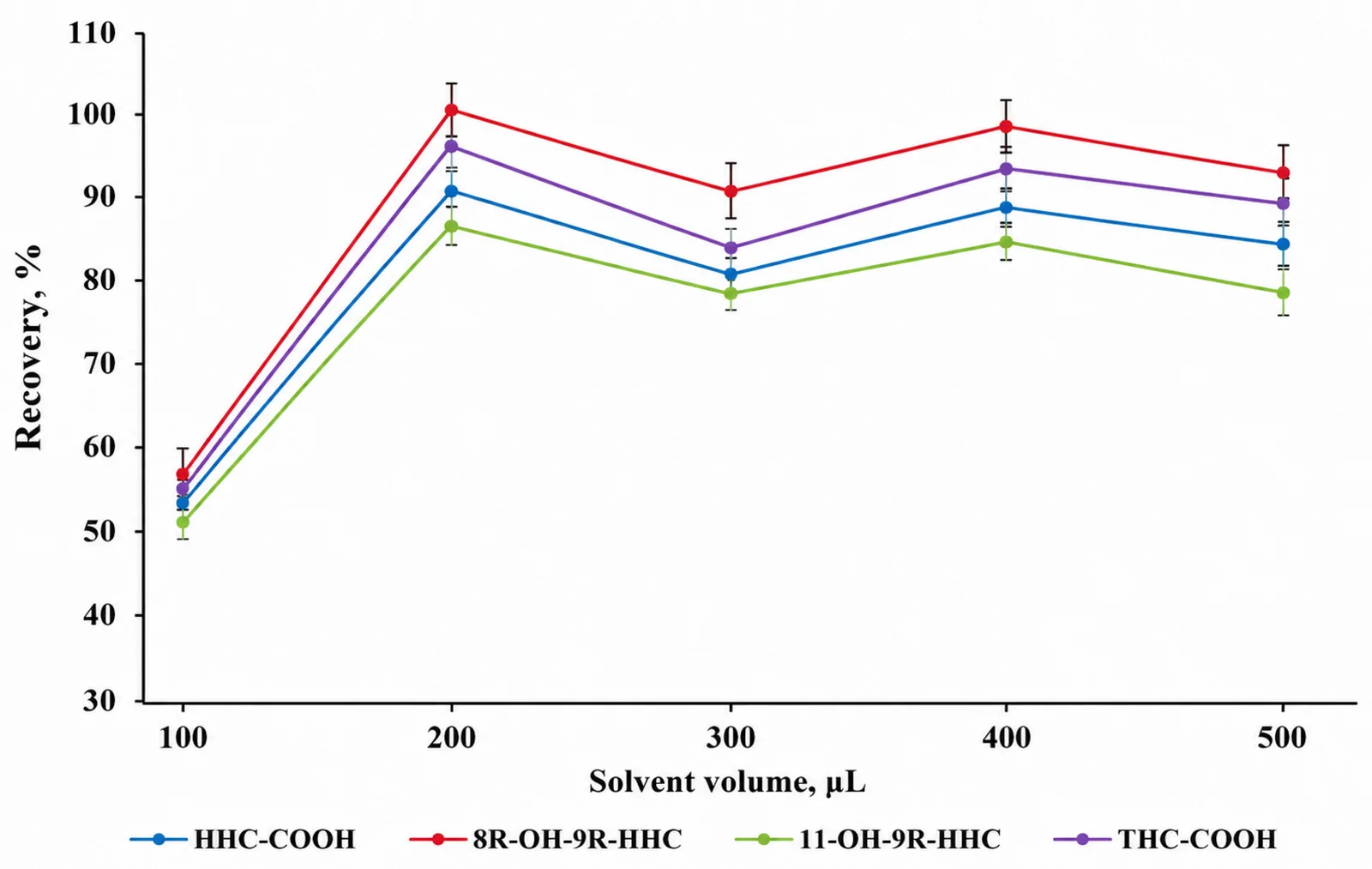
Optimization of the cyclohexane/ethyl acetate (9:1) volume. (Fixed variables: 100 µL urine, cyclohexane/ethyl acetate (9:1), 6 air-assisted extraction cycles, without NaCl). Data are expressed as mean ± SD (N = 4).

To determine the optimal solvent volume, different volumes of the cyclohexane/ethyl acetate (9:1) were evaluated: 100, 200, 300, 400 and 500 µL. The results are shown in Figure 4. The smallest tested volume (100 µL) proved insufficient for analyte extraction and, additionally, phase separation was difficult due to the small solvent volume. No substantial improvement in extraction efficiency was observed when increasing the extraction solvent volume from 200 to 500 µL. Considering the extraction performance and solvent consumption, 200 µL of the solvent mixture was selected as the optimal extraction volume for subsequent experiments.

#### 3.1.3. Study of the Air-Assisted Extraction Cycles

**Figure 5:**
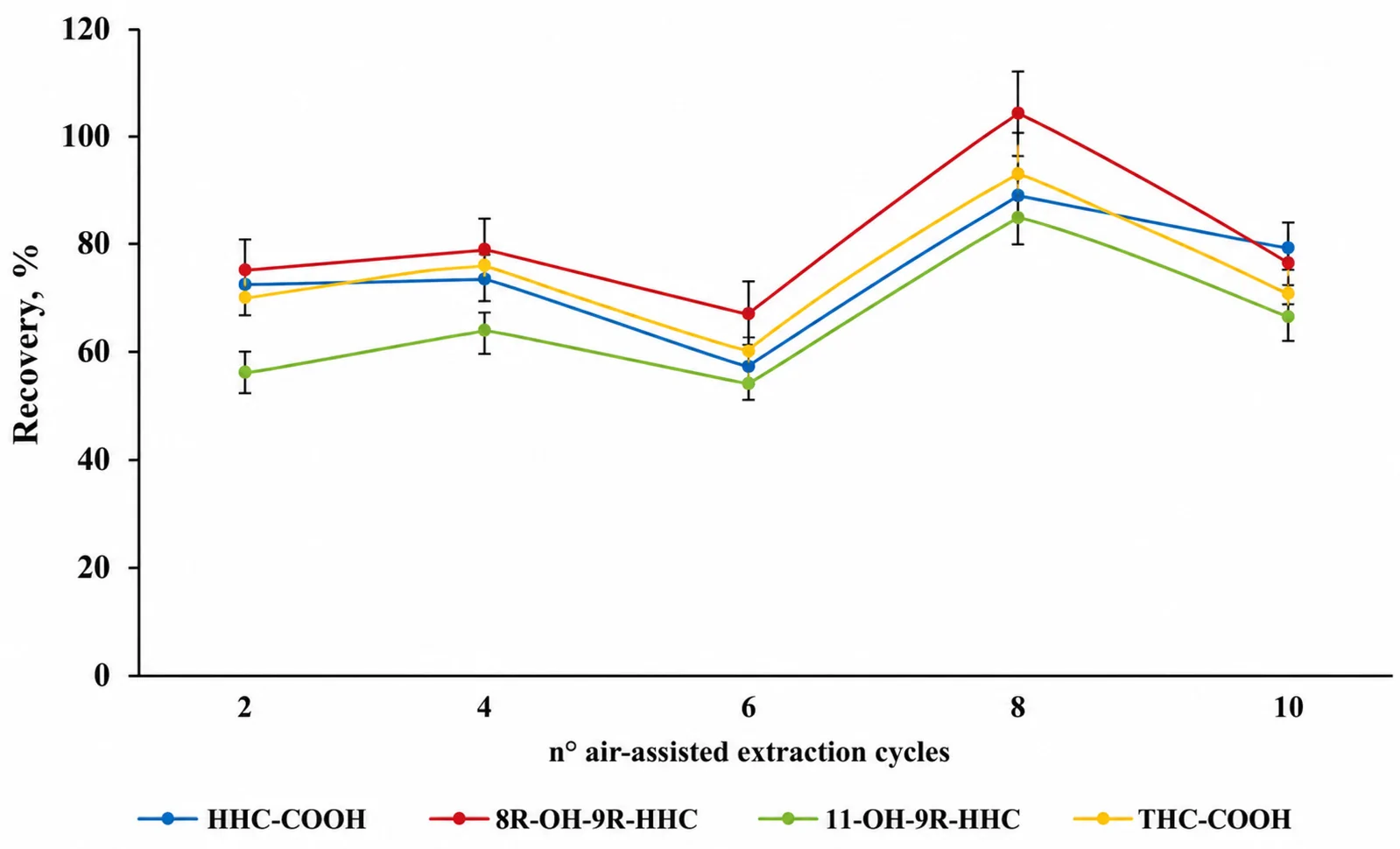
Optimization of the number of aspirations. (Fixed variables: 100 µL urine, 200 µL cyclohexane/ethyl acetate (9:1), without NaCl). Data are expressed as mean ± SD (N = 4).

The number of extraction cycles in AALLE refers to how many times the extraction solvent and sample solution are rapidly drawn into a Pasteur pipette and then expelled into a test tube. Increasing the number of these cycles can enhance extraction efficiency due to improved mixing. Therefore, the effect of the extraction cycles was evaluated within a range of 2 to 10 under constant experimental conditions. The results showed that the analytical signal increased with the number of cycles up to a maximum at eight cycles (Figure 5). Beyond this point, no further improvement was observed. Consequently, eight extraction cycles were selected as the optimal condition.

#### 3.1.4. Study of the Effect of Ionic Strength

The addition of salt can increase the ionic strength of the aqueous phase, potentially enhancing the transfer of analytes to the organic phase by reducing their solubility in water (salting-out effect). However, in microextraction techniques such as AALLME, this effect does not always lead to improved extraction efficiency, as physicochemical changes in the aqueous phase may occur. In particular, increased viscosity can reduce the diffusion coefficients of the analytes, thereby hindering mass transfer [26].

**Figure 6:**
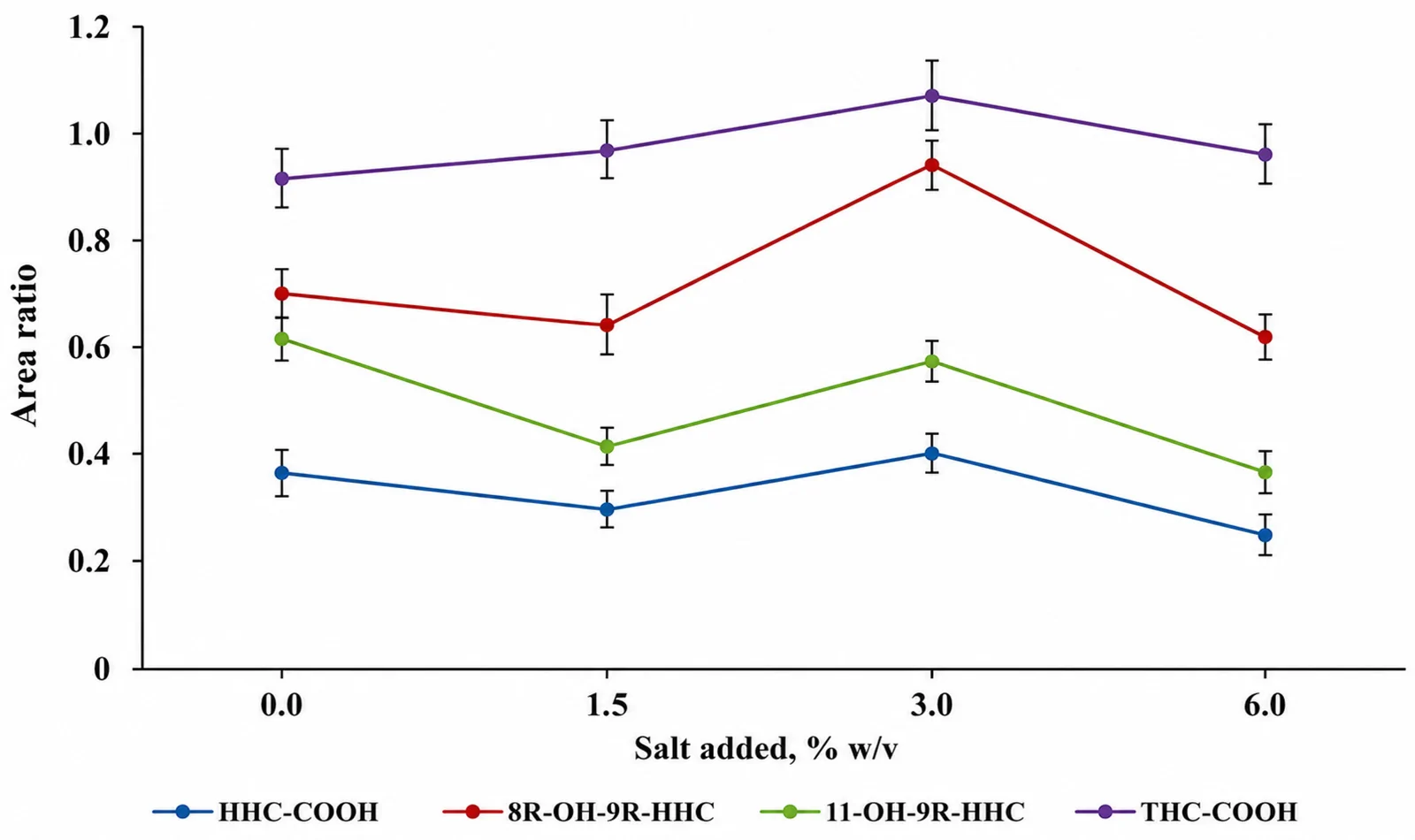
Optimization of the salting-out effect. (Fixed variables: 100 µL urine, 200 µL cyclohexane/ethyl acetate (9:1), 8 air-assisted extraction cycles). Data are expressed as mean ± SD (n= 4).

To evaluate this effect, different concentrations of sodium chloride (NaCl) (0, 1.5, 3, and 6%, *w*/*v*) were investigated (Figure 6). The results indicated that, for all four compounds studied, the addition of NaCl produced negligible changes in extraction efficiency. Since no significant improvement was observed, salt addition was not considered necessary and was therefore omitted from the final extraction procedure.

### 3.2. Validation of the Optimized Method

The validity of the method was assessed by determining the parameters described in Section 2.5. To carry out this study, blank urine samples were voluntarily provided by healthy laboratory researchers. All samples were anonymized prior to analysis and were confirmed to be drug-free before use. The samples were used exclusively for analytical method development and validation, and no personal identifying information was collected. This sample was spiked with known concentrations of the analytes under investigation.

#### 3.2.1. Selectivity

Selectivity was evaluated using blank urine samples from drug-free volunteers. Each sample was independently extracted and injected in triplicate. Data showed no interfering peaks at the retention times of the compounds under investigation.

#### 3.2.2. Sensitivity

LOD and LLOQ were evaluated at each concentration level using five replicate injections. The LOD and LLOQ values obtained for all analytes are presented in Table 3. The LOD corresponded to the lowest concentration at which the analytes could be reliably detected according to the identification criteria of the analytical method. The LLOQ was established as the lowest concentration fulfilling the predefined acceptance criteria for quantitative analysis, including precision (CV ≤ 20%) and accuracy within ±20% of the nominal concentration. Concentrations below the LLOQ (1 and 2.5 ng/mL) were evaluated during method development but did not consistently meet the criteria required for quantitative analysis. The detailed sensitivity data are provided in Table S1.

#### 3.2.3. Linearity

Linearity was assessed by preparing five independent calibration curves on different days. Each calibration level was injected in triplicate. The method exhibited a linear response over the concentration range of 5–70 ng/mL for all compounds, using calibration levels of 5, 10, 15, 30, 50 and 70 ng/mL, with correlation coefficients greater than 0.999 (Table 4). Although the validated calibration range was 5–70 ng/mL, samples exceeding the ULOQ may be reanalyzed following appropriate dilution, if required.

#### 3.2.4. Precision and Accuracy

Intraday accuracy and precision were evaluated at four QC levels (5, 10, 30 and 70 ng/mL). Four independent extractions were prepared for each QC level, and each extract was injected in triplicate within the same analytical run. Interday accuracy and precision were evaluated at the same four QC levels. Four independent extractions were prepared for each QC level on different days, and each extract was injected in triplicate.

Table 5 and Table 6 summarize the results obtained for the evaluation of intra- and inter-day precision and accuracy, expressed as BIAS (%) and coefficient of variation (CV, %). The data presented comply with the requirements set out in the relevant international guidelines (acceptance criteria of ±20% for the LLOQ and ±15% for the remaining concentration levels).

#### 3.2.5. Study of the Matrix Effect (ME), Process Efficiency (PE) and Extraction Recovery (RE)

Matrix effect, extraction recovery and process efficiency were evaluated at three QC levels (low, medium and high). Three independent extractions were prepared for each QC level, and each extract was injected in triplicate. The results of the ME, PE and RE are summarized in Table 7. The ME evaluated during method validation ranged from 92.8% to 115%, indicating a moderate but controlled influence of the sample matrix on the analytical response. Values below and above 100% suggest slight ion suppression and enhancement, respectively. However, the overall variability remained within acceptable limits, with CV values below 15% at all concentration levels, indicating good reproducibility of the matrix effect. Regarding PE, the values obtained during method validation ranged from 94.5% to 113.2%, indicating efficient overall sample preparation while accounting for both extraction recovery and matrix effects. Likewise, extraction recovery ranged from 95.6% to 110.6%, confirming efficient analyte extraction with good reproducibility. Although THC-COOH-d_3_ was used as the internal standard for all analytes, isotope-labelled analogues for the investigated HHC metabolites were not available in our laboratory at the time of the study. Therefore, analyte-specific compensation for matrix effects cannot be assumed and represents a limitation of the present work.

### 3.3. Greenness Assessment of the Analytical Procedure

In accordance with Green Analytical Chemistry (GAC) principles, the environmental sustainability of the proposed analytical workflow was evaluated using the AGREE (version software 1.0) [35] and ComplexMoGAPI (version software 1.0) [36] metrics. These tools enable a multidimensional assessment of analytical methods by considering factors such as reagent toxicity, solvent consumption, waste generation, sample preparation, instrumentation, and energy requirements.

**Figure 7:**
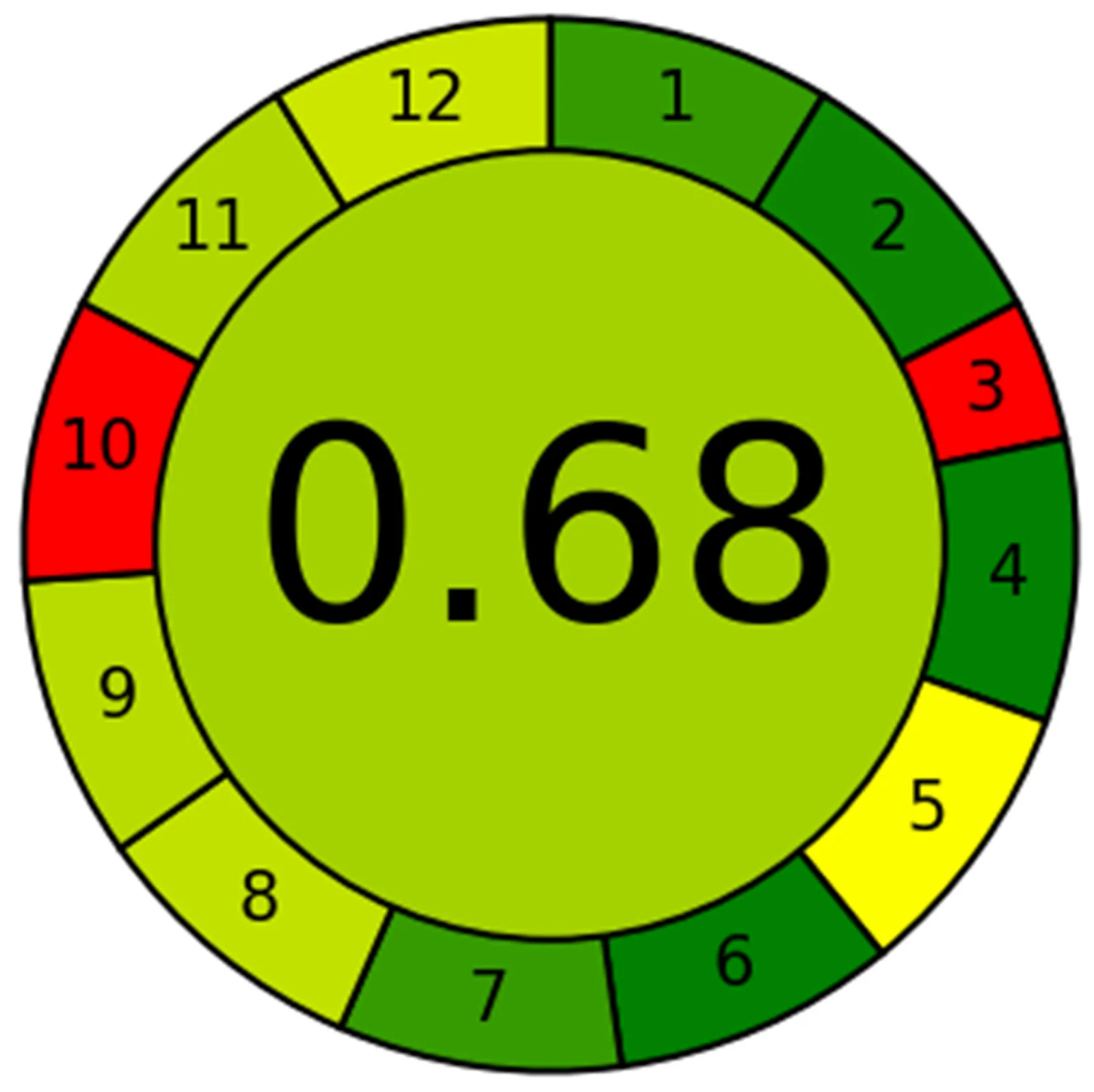
AGREE (version 1.0) assessment of sample preparation using AALLME-LC-MS/MS. The central value represents the overall greenness score (0-1), while the outer ring displays the performance for each of the 12 Green Analytical Chemistry principles. Green indicates good performance, yellow indicates moderate performance, and red indicated poor performance.

AGREE (Analytical GREEnness Metric Approach) is a software-based metric that evaluates analytical procedures according to the 12 principles of Green Analytical Chemistry and provides a final score ranging from 0 to 1 together with a visual pictogram representation. The proposed AALLME–LC–MS/MS method achieved an AGREE score of 0.68 (Figure 7), indicating a satisfactory greenness profile for a chromatographic method applied to forensic toxicology rather than a fully green analytical methodology. Although the method still requires LC–MS/MS instrumentation and the use of conventional laboratory reagents, its environmental performance is improved compared with conventional extraction procedures such as LLE and SPE, mainly due to the miniaturized air-assisted liquid–liquid microextraction procedure. This approach required only 200 µL of the extractant solvent mixture for the extraction of analytes from 100 µL of urine sample, thereby substantially reducing solvent consumption and waste generation. Additional positive contributions were related to the absence of derivatization steps, reduced analytical waste production, and the integration of sample preparation and analysis within a relatively streamlined workflow.

**Figure 8:**
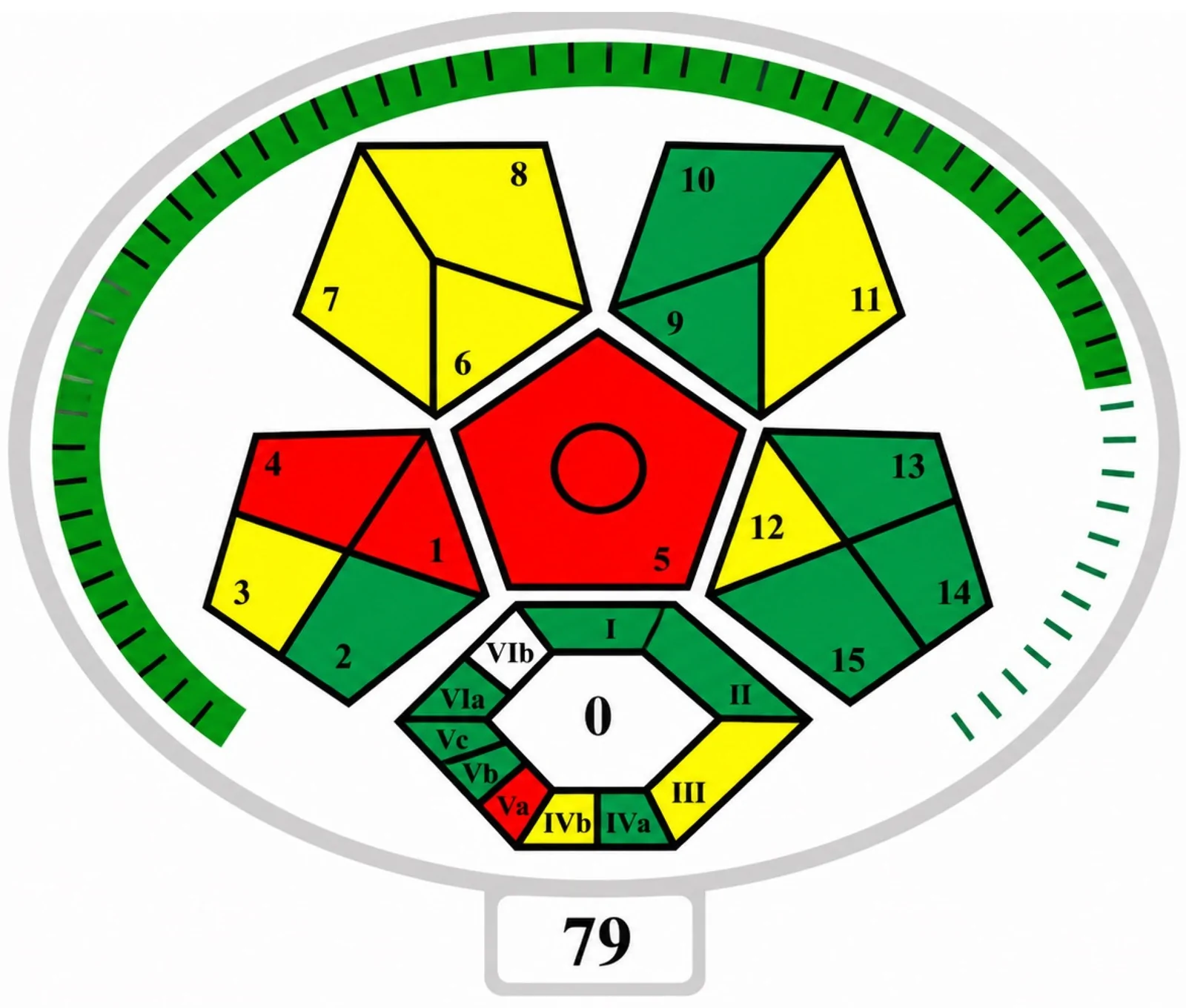
ComplexMoGAPI (version 1.0) assessment of sample preparation using AALLME-LC-MS/MS. The overall method greenness is represented by the final score (79/100) displayed at the bottom of the figure. The colored sectors represent the evaluation of the individual criteria included in the ComplexMoGAPI assessment. Green indicates good performance (high compliance with Green Analytical Chemistry principles), yellow indicates intermediate performance, and red indicates poor performance or criteria requiring improvement.

ComplexMoGAPI was additionally employed as a complementary greenness assessment tool. This metric integrates the principles of GAPI and MoGAPI and provides both graphical and numerical evaluation of the environmental impact of the analytical workflow. The proposed method achieved a ComplexMoGAPI score of 79 (Figure 8), indicating an improved greenness profile compared with conventional extraction procedures. The score reflects the reduced-scale sample preparation, low solvent requirements, and minimized chemical waste associated with the AALLME approach.

Nevertheless, both AGREE and ComplexMoGAPI present certain limitations when applied to forensic toxicology workflows. The obtained scores partially depend on predefined weighting systems and semi-quantitative classifications, which may introduce a degree of subjectivity into the assessment. In the present study, lower AGREE scores were mainly associated with the lack of in situ analysis, the energy requirements associated with LC–MS/MS instrumentation and the use of non-renewable organic solvents. With regard to this last point, although cyclohexane and ethyl acetate are volatile organic solvents with recognized hazards, their environmental impact was mitigated by the miniaturized extraction format and the very low solvent volumes employed. Similarly, the ComplexMoGAPI assessment penalized aspects related to offline sample collection and preparation, mandatory transport of biological specimens from forensic collection sites to the analytical laboratory, refrigerated storage conditions required to preserve sample integrity, and the need for an extraction step prior to chromatographic analysis.

These limitations are largely inherent to forensic toxicology applications involving biological matrices and highly sensitive confirmatory techniques. Despite these constraints, the proposed AALLME procedure minimizes solvent consumption, reduces chemical waste generation, and employs a miniaturized extraction strategy compared with conventional extraction methodologies. Overall, the greenness assessment indicates that the proposed AALLME–LC–MS/MS workflow represents a more sustainable alternative to conventional extraction procedures for cannabinoid biomarker determination in urine within the practical constraints of forensic toxicology analysis, rather than a fully green analytical methodology.

### 3.4. Application to Real Samples

The developed method was applied to eleven routine casework urine samples. These samples had originally been submitted for routine screening of drugs of abuse and tested positive for the THC main metabolite, THC-COOH.

The samples were subsequently reanalyzed using the method proposed in this study, and the results were compared with those obtained in the initial analysis.

Nine of the analyzed cases were positive for THC-COOH using the proposed method, while no detectable levels were observed for the remaining analytes. The measured concentrations ranged from 9.26 to 56.1 ng/mL, with one sample exceeding the upper limit of quantification (ULOQ, 70 ng/mL) and two samples falling below the lower limit of quantification (LLOQ).

When compared with the laboratory’s routine confirmatory LC–MS/MS method routinely employed for THC-COOH determination in urine (routine method calibration range: 5–50 ng/mL), the results showed good agreement, with percentage differences within ±15% for all comparable cases, indicating no significant bias between methods. This comparison was intended solely to assess the agreement between the proposed procedure and the routine analytical method for THC-COOH in authentic urine samples, since no authentic urine samples from confirmed HHC users were available for comparison.

Due to the recent emergence of semisynthetic cannabinoids in Europe, there is a clear need for analytical laboratories to develop and implement extraction and detection methods capable of reliably identifying and quantifying these new substances of concern. However, the currently available information on these compounds remains limited, highlighting the need for further research. Some authors have reported urinary concentrations of HHC metabolites; for instance, Helander A. et al. [6] described HHC-COOH levels ranging from 10 to 205 ng/mL, while Tsujikawa K. et al. [3] reported values between 9.4 and 13.4 ng/mL and up to 107 ng/mL. Additionally, Di Trana A. et al. [19] reported concentrations of 3.4–18.2 ng/mL for 8-OH-9(R)-HHC, 1.4–62.2 ng/mL for 11-OH-9(R)-HHC, and 3.4–6.8 ng/mL for HHC-COOH, with the highest concentrations observed for 11-OH-9(R)-HHC.

In the present study, no authentic urine samples from confirmed HHC consumers were available. In addition, glucuronide reference standards for the investigated HHC metabolites were also unavailable. Therefore, direct experimental confirmation of their deconjugation could not be performed. Nevertheless, the alkaline hydrolysis conditions employed are routinely used in our laboratory for cannabinoid analysis. As an additional assessment of the hydrolysis step, the MRM transitions corresponding to THC-COOH-glucuronide were monitored in this study after hydrolysis. No signals corresponding to these transitions were detected in the analyzed urine samples. Although this observation supports the effectiveness of the selected hydrolysis conditions, direct confirmation of complete deconjugation of HHC metabolites was not possible in the absence of glucuronide reference standards. Future studies including authentic HHC glucuronide standards should further evaluate the hydrolysis efficiency for these metabolites. Despite these limitations, given the growing relevance and potential public health impact of these substances, a proactive approach was adopted to develop and validate an analytical method intended to support their determination in urine. The proposed method demonstrated efficient extraction using low solvent volumes and provided satisfactory validation results for quantitative analysis.

## 4. Conclusions

AALLME was developed as a more sustainable sample preparation approach for the determination of THC-COOH and selected HHC metabolites in urine. Compared with conventional liquid–liquid microextraction procedures, the proposed method eliminates the use of halogenated solvents and reduces organic solvent consumption while providing satisfactory analytical performance.

The method was successfully validated according to international guidelines, demonstrating satisfactory sensitivity, precision and accuracy for the quantitative determination of the investigated analytes in fortified urine samples.

The applicability of the method was demonstrated through the analysis of authentic THC-COOH-positive urine samples, which showed good agreement with the laboratory’s routine confirmatory LC–MS/MS method. Although no authentic HHC-positive urine samples were available for evaluation, the satisfactory validation results obtained in fortified urine samples indicate that the proposed method may support the determination of HHC metabolites. However, its performance should be confirmed in future studies including authentic urine samples from confirmed HHC users.

## 5. Limitations

A limitation of the present study is that authentic glucuronide reference standards for the investigated HHC metabolites were not available. Consequently, direct experimental confirmation of the efficiency of the alkaline hydrolysis step could not be performed. Nevertheless, the hydrolysis conditions employed are routinely used in our laboratory for cannabinoid analysis, and qualitative monitoring of THC-COOH-glucuronide showed no residual signals after hydrolysis, supporting the suitability of the hydrolysis procedure under the conditions employed. Furthermore, although the method was successfully validated using fortified urine samples, its applicability to authentic HHC-positive urine samples could not be fully assessed due to the limited availability of confirmed positive specimens. Finally, a single isotope-labelled internal standard (THC-COOH-d_3_) was used for all analytes because isotope-labelled analogues of the investigated HHC metabolites were not available in our laboratory at the time of the study. Although suitable for the purposes of this work, analyte-specific isotope-labelled internal standards would provide more effective compensation for recovery and matrix effects.

## 6. Future Perspectives and Practical Implications

Future studies should evaluate the proposed methodology using a larger number of authentic HHC-positive urine samples and authentic glucuronide reference standards to directly assess the efficiency of the alkaline hydrolysis step. The applicability of the AALLME procedure may also be extended to other emerging semisynthetic cannabinoids as additional analytical standards become available. From a practical perspective, the proposed method is intended for implementation in forensic and clinical toxicology laboratories equipped with LC–MS/MS instrumentation for routine confirmatory drug testing. Although a formal cost analysis was beyond the scope of this study, the simplified extraction procedure, reduced solvent consumption, and the absence of SPE cartridges or disperser solvents may contribute to lowering consumable costs and laboratory waste.

## Figures and Tables

**Table 1:** Gradient used for semisynthetic cannabinoids (equation time: equilibration time).

Time, min	Flow, mL/min	%A	%B
0	0.35	40	60
1	0.35	30	70
4	0.35	28	72
5	0.35	25	75
6	0.35	20	80
7	0.35	15	85
11.0	0.35	10	90
12	0.35	40	60
13	0.35	40	60
+3 min equation time	0.35	40	60

**Table 2:** Information on the compounds analyzed (t_R_, retention time; CE, collision energy). MRM transitions for THC-COOH-glucuronide, included in the routine laboratory acquisition method, were used exclusively for qualitative monitoring of hydrolysis efficiency and were not part of the validated quantitative method described in this work.

Analyte Name	t_R_, min	Precursor Ion	Quantifier Transition (CE)	Qualifier Transition (CE)
8-OH-9(R)-HHC	4.4	333	333–259 (25)	333–315 (20)
				333–193 (20)
				333–135 (20)
HHC-COOH	6.2	347	347–193 (20)	347–329 (20)
				347–121 (20)
				347–301 (20)
11-OH-9(R)-HHC	6.4	333	333–123 (40)	333–315 (20)
				333–259 (30)
				333–193 (10)
THC-COOH	6.6	345	345–327 (20)	345–193 (20)
				345–299 (20)
THC-COOH-d3	6.5	348	348–330 (20)	348–196 (20) 348–330 (20)
THC-COOH glucuronide	5.5	521	521–345.9 (20)	521–181.6 (20) 521–119 (35)

**Table 3:** LLOQ and LOD values for the analytes studied.

	8-OH-9(R)-HHC	HHC-COOH	11-OH-9(R)-HHC	THC-COOH
LLOQ, ng/mL	5	5	5	5
LOD, ng/mL	2.5	2.5	2.5	2.5

**Table 4:** Calibration curves.

	Intercept	Slope	r^2^
HHC-COOH	0.02839191	0.035691787	0.9992
8-OH-9(R)-HHC	0.10430528	0.06704836	0.9997
11-OH-9(R)-HHC	−0.405640387	0.10585836	0.9996
THC-COOH	0.14834702	0.210003232	0.9993

**Table 5:** Intra-day study for the compounds studied.

	Concentration, ng/mL	BIAS ± Error (%)	Precision, CV (%)
8-OH-9(R)-HHC	5	5.51 ± 4	5.29
	10	−4.49 ± 2.17	7.18
	30	−1.73 ± 0.91	2.92
	70	−0.53 ± 2.15	5.29
HHC-COOH	5	4.38 ± 5.58	10.04
	10	1.64 ± 1.97	6.14
	30	8.42 ± 1.84	3.79
	70	−0.68 ± 1.17	2.88
11-OH-9(R)-HHC	5	7.40 ± 6.93	8.14
	10	2.95 ± 4.17	9.06
	30	−4.44 ± 1.61	4.77
	70	−0.81 ± 2.42	5.47
THC-COOH	5	5.35 ± 3.95	3.89
	10	5.48 ± 2.67	7.59
	30	−3.33 ± 3.29	7.60
	70	−3.04 ± 3.15	7.28

**Table 6:** Inter-day study for the compounds studied.

	Concentration, ng/mL	BIAS ± Error (%)	Precision, CV (%)
8-OH-9(R)-HHC	5	16.2 ± 2.9	4.1
	10	3.97 ± 1.93	3.71
	30	0.28 ± 3.48	6.93
	70	2.05 ± 0.70	1.38
HHC-COOH	5	9.46 ± 7.81	6.3
	10	9.15 ± 2.50	4.59
	30	0.98 ± 1.81	3.60
	70	1.03 ± 0.76	1.69
11-OH-9(R)-HHC	5	12.2 ± 3.5	7.90
	10	9.9 ± 2.42	6.14
	30	6.54 ± 8.58	7.11
	70	3.85 ± 2.56	4.92
THC-COOH	5	7.81 ± 5.3	9.6
	10	0.69 ± 4.46	8.87
	30	3.06 ± 1.34	2.61
	70	0.70 ± 0.87	1.74

**Table 7:** ME, PE and RE for the cannabinoids at three concentrations (CV_ME_: matrix effect variability).

	Concentration, ng/mL	ME (%)	CV_ME_	PE (%)	RE(%)
8-OH-9(R)-HHC	5	111.5	7.69	113.2	110.6
	30	99.3	6.9	97.2	97.8
	70	115.0	5.73	110.4	102.6
HHC-COOH	5	113.9	11.5	96.2	98.9
	30	99.9	2.69	104.1	104.2
	70	112.2	3.22	100.8	102.3
11-OH-9(R)-HHC	5	92.8	6.6	102.5	102.3
	30	93.7	4.97	96.6	95.6
	70	109.6	1.45	103.4	103.4
THC-COOH	5	100.5	6.1	103.4	101.3
	30	98.4	1.7	94.5	96.1
	70	109.2	5.6	102.2	106.5

## Data Availability

The original contributions presented in this study are included in the article/Supplementary Material. Further inquiries can be directed to the corresponding author.

## Supplementary Materials

The following supporting information can be downloaded at: https://www.mdpi.com/article/10.3390/jox16040143/s1. Table S1: Statistical study of LOD and LLOQ (CV, coefficient of variation).
