# Supplementary material for: A Sustainable Air-Assisted Liquid–Liquid Microextraction Procedure Coupled to Liquid Chromatography–Tandem Mass Spectrometry for the Determination of 11-Nor-9-carboxy-Δ9-tetrahydrocannabinol and Hexahydrocannabinol Metabolites in Urine
Source: J Xenobiot. 2026 Aug 1;16(4):143. doi: 10.3390/jox16040143 (PMC13514403; doi:10.3390/jox16040143)
Supplement: Supplementary file 1 [file jox-16-00143-s001.zip › jox-4386181-supplementary.pdf]

Supplementary Materials: A Sustainable Air-Assisted Liquid–Liquid Microextraction Procedure Coupled to Liquid Chromatography–Tandem Mass Spectrometry for the Determination of 11-Nor-9-carboxy-Δ^9-tetrahydrocannabinol and Hexahydrocannabinol Metabolites in Urine

Pamela Cabarcos-Fernández, Sara Odoardi, Serena Mestria, Valeria Valentini, Giulia Biosa, Ana María Bermejo-Barrera and Sabina Strano-Rossi

Table S1. Sensitivity study performed during method validation (CV, coefficient of variation).

	Concentration, ng/mL	S/N	Bias (%)	Accuracy (%)	CV (%)	Status
**HHC-COOH**	**10**	36.6	-0.33	99.7	18.8	QC level
	**5**	17.9	6.27	106.2	7.8	**Accepted LLOQ**
	**2.5**	5.77	-2.7	97.3	51.3	Below LLOQ
	**1**	5.11	50.6	49.3	72.8	Below LLOQ
**8-OH-9(R)-HHC**	**10**	29	0.50	100.5	5.77	QC level
	**5**	14.1	19.4	119.4	9.71	**Accepted LLOQ**
	**2.5**	6.1	46.8	146.8	25.0	Below LLOQ
	**1**	2.48	103.2	203.3	12.8	Below LLOQ
**11-OH-9(R)-HHC**	**10**	16.4	12.3	101.9	13.8	QC level
	**5**	8.3	12.9	109.0	3.12	**Accepted LLOQ**
	**2.5**	4.19	131.7	231.7	11.9	Below LLOQ
	**1**	1.87	314	414	62.6	Below LLOQ
**THC-COOH**	**10**	42.7	1.44	101.4	24.2	QC level
	**5**	31.4	14.1	120.0	12.8	**Accepted LLOQ**
	**2.5**	12.6	73.2	173.2	17.9	Below LLOQ
	**1**	5.64	242.7	342.7	2.4	Below LLOQ

Note: Concentrations of 1 and 2.5 ng/mL were evaluated during the sensitivity assessment but were not accepted as LLOQ because the predefined criteria for quantitative analysis (accuracy and/or precision) were not consistently fulfilled.
